# Necrotizing Uterine Infection After Dilation and Evacuation: A Case Report

**DOI:** 10.7759/cureus.109054

**Published:** 2026-05-17

**Authors:** Kayla S Menendez, Lauren Thaxton, Leilah Zahedi-Spung, Marisa Moroney, Megan Masten

**Affiliations:** 1 Obstetrics and Gynecology, University of Colorado Anschutz, Aurora, USA; 2 Complex Family Planning &amp; Maternal Fetal Medicine, University of Colorado Anschutz, Aurora, USA; 3 Gynecologic Oncology, University of Colorado Anschutz, Aurora, USA; 4 Complex Family Planning, MaineHealth Maine Medical Center Portland, Portland, USA

**Keywords:** dilation and evacuation, hysterectomy, immunosuppression, maternal sepsis, previable pprom, septic abortion, uterine necrosis

## Abstract

Previable premature prelabor rupture of membranes (PPROM) can lead to uterine infection, and sepsis with uterine necrosis is a rare complication. Prompt source control with abortion care if infection develops is an important component of management. We report the case of uterine necrosis in an immunosuppressed patient. A 25-year-old gravida 5, para 1-0-3-1, on immunosuppressive medications for systemic lupus erythematosus, presented at 15 weeks with PPROM and sepsis. She was treated with broad-spectrum intravenous antibiotics and dilation and evacuation (D&E) for a septic abortion. Her clinical status did not improve postoperatively, and workup, including imaging, revealed an ascending infection with a uterine source. After counseling, she underwent total abdominal hysterectomy with uterine necrosis confirmed on pathologic examination. Endometrial cultures grew multiple organisms sensitive to the antibiotic regimen. The patient recovered and was discharged on postoperative day five. Uterine necrosis can be a severe complication of septic abortion and presents as worsening pain and sepsis despite uterine evacuation. Non-toxin and non-gas-producing bacteria can be implicated. A high index of suspicion is required for appropriate treatment, particularly for immunosuppressed patients. Definitive surgical management of uterine necrosis with hysterectomy should be considered.

## Introduction

Uterine necrosis is a rare complication that results from severe endometritis [1]. Uterine necrosis occurs from ischemia of myometrial tissue, most commonly due to vascular compromise [2]. Reduced or occluded uterine arterial inflow leads to tissue ischemia and subsequent necrosis of the myometrium [2]. Endometritis and intra-amniotic infections are the most common causes of maternal sepsis [3]. Intra-amniotic infections are typically polymicrobial. It is uncommon to isolate a single causative bacterium, with the exception of toxin-producing bacteria, such as Clostridium perfringens and Group A Streptococcus (GAS), known to cause lethal necrotizing infections [4]. In high-income countries, most maternal infectious deaths are caused by C. perfringens or GAS [4]. Complications of infectious uterine necrosis include peritonitis, pelvic abscess, sepsis, and death [5]. If a patient is pregnant, prompt uterine evacuation is required for source control. Hysterectomy may be recommended for adequate source control in a postpartum setting if uterine necrosis is suspected [6]. We present a case of uterine necrosis in an immunosuppressed patient necessitating hysterectomy following septic abortion, and requiring dilation and evacuation (D&E) for previable premature prelabor rupture of membranes (PPROM).

## Case presentation

A 25-year-old gravida 5, para 1-0-3-1 woman presented at 15 weeks and five days of gestation. Her medical history was significant for systemic lupus erythematosus on immunosuppressant medications (azathioprine and hydroxychloroquine), and antiphospholipid antibody syndrome. Four days prior, she had presented to an outside hospital with fever and abdominal pain and was diagnosed with pyelonephritis. She received one dose of intravenous ceftriaxone and was discharged with oral cefdinir. The following day, she reported fluid leakage and presented to another hospital. A ROM Plus® test (Clinical Innovations, LLC, Utah, USA) was negative. A four-point rupture-of-membrane examination was not performed, and ultrasonography demonstrated normal amniotic fluid with a maximum vertical pocket of 3.3 cm. She was discharged with instructions to continue oral antibiotics for outpatient management of pyelonephritis.

Over the next two days, she continued to experience intermittent leakage of clear fluid. She then presented to an outside hospital with clear, blood-tinged fluid and abdominal pain and was subsequently transferred to our institution for higher-level care.

On arrival, she reported worsening abdominal pain and had vital sign abnormalities, including tachycardia to 120 beats/minute (reference range: 60-100 beats/minute), hypotension (80/50 mmHg (reference value: 120/80 mmHg), and was febrile to 38.3°C (reference range: 36.5-37.5°C). Examination revealed fundal tenderness. Pelvic examination showed copious yellow, cloudy fluid with pooling that was nitrazine-positive. Laboratory evaluation demonstrated leukopenia with a white blood cell count of 2.8 × 10^9^/L (reference range: 4.0-11.1 × 10^9^/L) and a lactate level of 2.3 mmol/L (reference range: 0.5-2.2 mmol/L). Blood and urine cultures were obtained. She was admitted with sepsis secondary to PPROM. She received intravenous fluids (IV) and broad-spectrum IV antibiotics with ampicillin, gentamicin, and metronidazole.

After counseling, the patient opted for D&E, which was performed later that day. Intraoperative findings were notable for yellow-green amniotic fluid pooling in the posterior vaginal vault, cervical dilation of 2 cm, and a firm, irregular-appearing mass on the anterior aspect of the cervix that grossly enlarged it. A rectovaginal examination revealed no parametric nodularity. A biopsy of the mass was performed intraoperatively, and hemostasis was noted.

**Figure 1:**
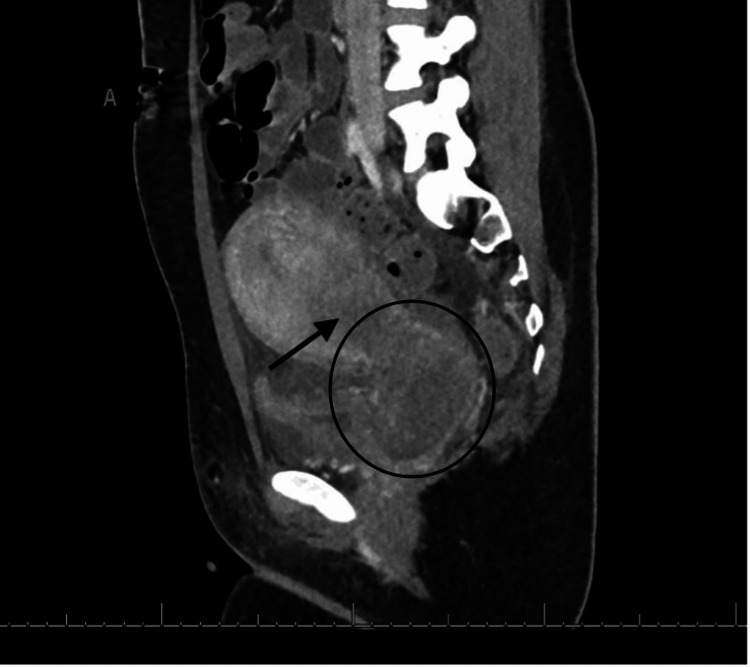
Contrast-enhanced computed tomography findings of cervical and uterine infection Saggital image shows cervical enlargement (circled), areas of hypoenhancement, necrosis, and ascending uterine involvement (arrow).

Postoperatively, the patient initially showed clinical improvement and was afebrile with normal vital signs and lactate levels. Approximately 24 hours after the D&E and the last febrile episode, broad-spectrum antibiotics were narrowed to oral doxycycline and metronidazole to treat the presumed endometritis. On postoperative day two, she reported worsening abdominal pain, nausea, and bloating. Objective signs of worsening infection developed, including hypotension, fever, and ongoing leukopenia. Blood cultures were collected. IV antibiotics were resumed and broadened to piperacillin-tazobactam, clindamycin, and linezolid due to concern for possible GAS infection, given the severity of illness. Computed tomography (CT) of the abdomen and pelvis showed heterogeneous enhancement of the uterus, with hypoenhancement of the lower uterine segment and cervix. On CT, her cervix was enlarged, measuring 7.0 cm × 5.3 cm (Figure 1).

**Figure 2:**
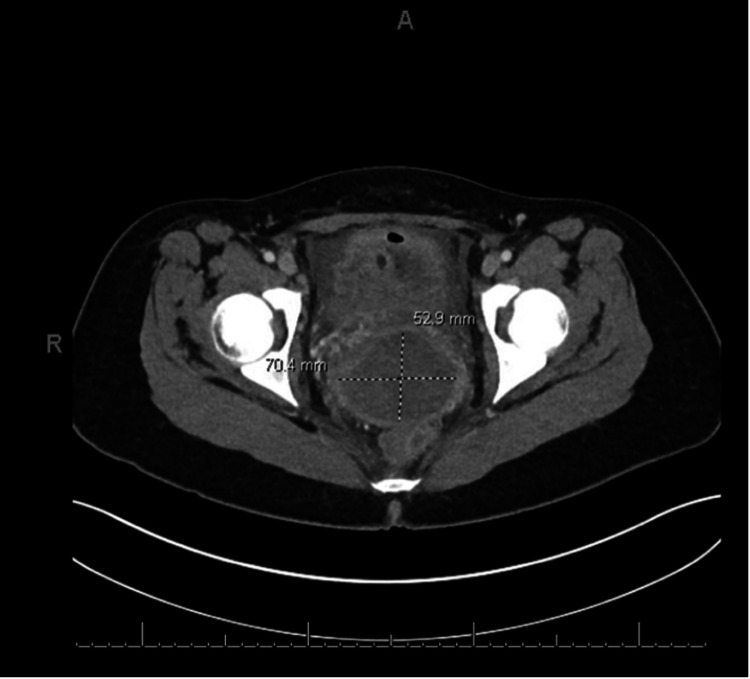
Contrast-enhanced computed tomography findings of cervical and uterine infection Axial image shows measurements of enlarged cervix 52.9 mm x 70.4 mm.

There were also findings concerning for cervical necrosis and ascending infection to the uterus (Figure 2).

Biopsy specimens obtained during the D&E demonstrated extensive necrosis and inflammation. 

The patient was transferred to the intensive care unit (ICU) for septic shock and remained febrile and hypotensive despite broad-spectrum antibiotic therapy. Given her worsening condition and suspected uterine necrosis, the patient was counseled regarding definitive source control with hysterectomy. The patient desired future fertility if possible, though was understanding of the life-threatening circumstances.

**Figure 3:**
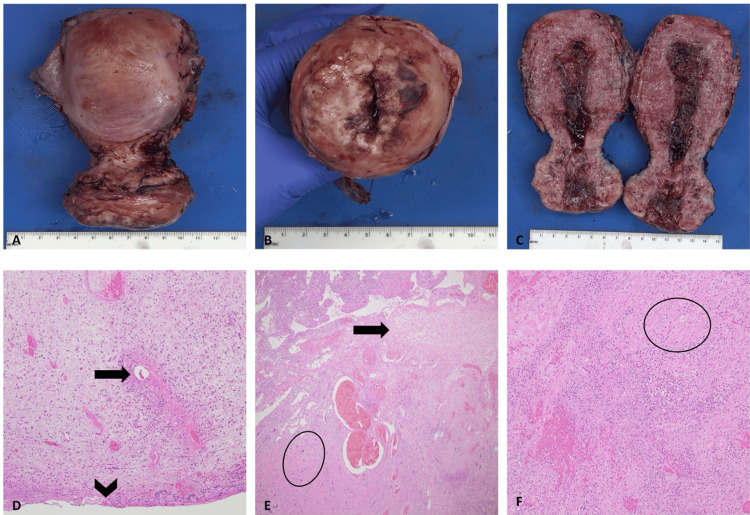
Gross and histopathologic findings following hysterectomy (A) Anterior aspect of hysterectomy specimen; (B) Bulky cervix with regions of ulceration and fibrinopurulent exudate; (C) Bivalved uterus demonstrating a hemorrhagic endometrial cavity without a discrete mass; (D) Cervix showing transition from normal stratified squamous epithelium to denuded mucosa (arrowhead) and vessels with fibrinoid necrosis (arrow) (hematoxylin and eosin (H&E, ×100); (E) Endometrium with necrosis (arrow) and implantation-site trophoblast invading the myometrium (circle) (H&E, ×40); (F) Myometrium with extensive acute inflammation and regions of coagulative necrosis (circle) (H&E, ×100).

After counseling, she agreed that definitive surgery was the best course of action and proceeded to the operating room for exploratory laparotomy, total abdominal hysterectomy, and bilateral salpingectomy for suspected GAS infection due to CT imaging findings and clinical worsening despite broad-spectrum IV antibiotics and initial source control with D&E. Intraoperative findings were notable for the cervix circumferentially enlarged to 8 cm, firm, and normal surrounding parametria. On speculum examination, the cervix appeared necrotic with dusky central tissue and purulent areas. The uterus was enlarged and diffusely boggy (Figure 3).

The patient tolerated the procedure well and showed clinical improvement postoperatively. Blood culture results were negative. Endometrial cultures grew Enterococcus faecalis, Enterococcus faecium, Streptococcus anginosus, and Prevotella bivia. She was discharged on postoperative day four and continued oral metronidazole and doxycycline for a total of 14 days. The final pathological examination revealed a partially denuded cervix with extensive necrosis. Sections from the cervix, lower uterine segment, and endomyometrium showed extensive acute and chronic inflammation with associated necrosis. Much of the myometrium appeared necrotic, with viable myometrium that was more prominently concentrated in the outer half. These findings were compatible with necrotizing infection.

## Discussion

Uterine necrosis is rare and potentially fatal. The most common infectious causes of uterine necrosis include GAS, Escherichia coli, C. perfringens, and Fusobacterium necrophorum [1]. To our knowledge, this represents a rare case of infectious uterine necrosis without any identified toxin or gas-forming microbiology, identified following D&E performed for PPROM [1]. Postpartum patients have been reported to have a 20-fold higher risk of GAS infection than nonpregnant patients and have an increased risk of mortality [7]. Chorioamnionitis and PPROM were important risk factors for this patient. Postpartum or post-abortion endometritis can be managed with antibiotics if diagnosed at an early stage. First-line therapy involves beta-lactam antibiotics combined with clindamycin or linezolid to decrease GAS toxin synthesis [8]. Interestingly, the bacteria isolated from the endometrium in our case were not associated with toxin formation or uterine necrosis. The patient’s immunocompromised status was likely due to immunosuppressive lupus medications that contributed to her worsening clinical picture, particularly in the presence of non-toxin or non-gas-producing bacteria.

Pelvic ultrasonography is an initial diagnostic test that can reveal evidence of uterine necrosis [9]. The uterine cavity typically expands and exhibits multiple echogenic foci accompanied by acoustic shadowing [10,11]. Diagnosis may require further evaluation with CT or magnetic resonance imaging [10,11]. CT can demonstrate the presence of gas in the myometrium, lack of myometrial enhancement after contrast administration, uterine enlargement, and free intraperitoneal fluid. However, given the rarity of these cases, no pathognomonic radiological features have been established [10-12]. In our case, CT imaging was instrumental in raising concern for uterine necrosis and aided in clinical decision-making for treatment and in the identification of a source while awaiting culture results. Intraoperative examination findings during D&E, with an enlarged and dusky-appearing cervix, also raised a clinical suspicion of necrosis.

Given the limited number of reported cases of uterine necrosis, no standardized treatment protocol has been established [1]. However, owing to the life-threatening nature of the disease, emergency hysterectomy with broad-spectrum antibiotic therapy is recommended [12]. The patient improved clinically once source control was performed via total hysterectomy.

Symptoms of inflammation may be attenuated in immunosuppressed patients, potentially delaying recognition of uterine pathology [13]. This altered inflammatory response in immunosuppressed patients may result in diminished clinical findings, making early diagnosis more challenging [13]. A high index of suspicion is required to facilitate early recognition and management of uterine necrosis, including in patients undergoing D&E for PPROM.

Finally, timely uterine evacuation is critical for patients with PPROM complicated by sepsis. According to the American College of Obstetricians and Gynecologists, if PPROM occurs before 24 weeks of gestation, immediate delivery with induction of labor or D&E should be offered [14]. If sepsis occurs in the setting of PPROM, antibiotic administration and uterine evacuation with induction of labor or D&E should be followed [15]. Due to recent abortion care restrictions, patients seeking care in the setting of PPROM have limited access to uterine evacuation [16]. A comparative study of PPROM before and after the Texas Senate Bill 8 (SB8), which banned abortion care after a fetal heartbeat can be detected, found that women post-SB8 were more likely to develop adverse outcomes (35.4% vs. 22.9%) and sepsis (29.2% vs. 9.4%), with longer intervals from membrane rupture to delivery (6.5 vs. 3 days) due to state-mandated expectant management [16]. Fetal outcomes showed no improvement, with 96% of patients experiencing stillbirth or neonatal death in a Texas series [16].

Expectant management significantly increased maternal morbidity compared with abortion care. In a multicenter retrospective cohort study, expectant management was associated with 3.47 times the odds of composite maternal morbidity (60.2% vs. 33.0%) after adjusting for confounders, including higher rates of chorioamnionitis (38.0% vs. 13.0%) and postpartum hemorrhage (23.1% vs. 11.0%) [14]. ICU admissions and unplanned hysterectomies occurred only in the expectant management group [14]. A systematic review confirmed an increased risk of infection, hemorrhage, and death with expectant management [17]. This case underscores the importance of timely uterine evacuation, and the benefit of being able to offer D&E to all patients and particularly in immunosuppressed individuals. In this case, despite timely intervention, infectious sequelae progressed, ultimately requiring hysterectomy for definitive source control.

## Conclusions

This case demonstrates that severe endometritis and uterine necrosis may occur even with non-toxin and non-gas-producing bacteria, and may be especially difficult to recognize in immunosuppressed patients. Patients with PPROM should be counseled and offered prompt uterine evacuation with dilation and evacuation due to the risk of infection, labor augmentation, or expectant management. Worsening pain or sepsis despite uterine evacuation may indicate retained tissue, severe infection or uterine necrosis. Clinicians must consider definitive surgical management with hysterectomy for source control when uterine necrosis is suspected.

## References

[1] Yu T (2024). Minimally invasive treatment of uterine necrosis with favorable outcomes: an uncommon case presentation and literature review. BMC Womens Health.

[2] Poujade O, Ceccaldi PF, Davitian C (2013). Uterine necrosis following pelvic arterial embolization for post-partum hemorrhage: review of the literature. Eur J Obstet Gynecol Reprod Biol.

[3] Nieuwoudt C, White SE, Heine RP, Widelock TM (2024). Maternal sepsis. Clin Obstet Gynecol.

[4] Jacques L, Kelly B, Soehl J (2024). Peripartum uterine clostridial myonecrosis: a report of two fatal cases. WMJ.

[5] Guo XM, Runge M, Miller D, Aaby D, Milad M (2020). A bundled intervention lowers surgical site infection in hysterectomy for benign and malignant indications. Int J Gynaecol Obstet.

[6] Olp RJ, Chamales IA, Schmiedecke SS (2020). A case study of puerperal group A streptococcal infection complicated by toxic shock syndrome. AJP Rep.

[7] Hamilton SM, Stevens DL, Bryant AE (2013). Pregnancy-related group a streptococcal infections: temporal relationships between bacterial acquisition, infection onset, clinical findings, and outcome. Clin Infect Dis.

[8] Dharia S, Shah S, Kissinger M, Sanders A, Singh G (2023). Group A streptococcal endometritis and toxic shock causing septic pelvic thrombophlebitis and septic pulmonary emboli. BMJ Case Rep.

[9] Ruiz Sánchez E, Peinado Rodenas J, Gil Martínez-Acacio L, Arones Collantes M, Villar García M, García de la Torre JP, Amezcua Recover AN (2021). Uterine necrosis. A rare complication of embolisation due to post-partum haemorrhage. J Gynecol Obstet Hum Reprod.

[10] Benkirane S, Saadi H, Serji B, Mimouni A (2017). Uterine necrosis following a combination of uterine compression sutures and vascular ligation during a postpartum hemorrhage: a case report. Int J Surg Case Rep.

[11] Pirard C, Squifflet J, Gilles A, Donnez J (2002). Uterine necrosis and sepsis after vascular embolization and surgical ligation in a patient with postpartum hemorrhage. Fertil Steril.

[12] Fouad A, Bouab M, Youssouf N, Lamrissi A, Fichtali K, Bouhya S (2022). Uterine necrosis simulating a textiloma: a case report. Int J Surg Case Rep.

[13] Fishman JA (2011). Infections in immunocompromised hosts and organ transplant recipients: essentials. Liver Transpl.

[14] Siegler Y, Weiner Z, Solt I (2020). ACOG practice bulletin no. 217: prelabor rupture of membranes. Obstet Gynecol.

[15] Sklar A, Sheeder J, Davis AR, Wilson C, Teal SB (2022). Maternal morbidity after preterm premature rupture of membranes at <24 weeks' gestation. Am J Obstet Gynecol.

[16] Ghafir D, Fahl E, Ukoh N, Chen HY, Blackwell SC, Gutierrez J, Stafford IA (2026). Maternal morbidity following periviable prelabor rupture of membranes after Texas Senate Bill 8. Am J Perinatol.

[17] Battarbee AN, Osmundson SS, McCarthy AM, Louis JM (2024). Society for Maternal-Fetal Medicine consult series #71: management of previable and periviable preterm prelabor rupture of membranes. Am J Obstet Gynecol.

